# Establishment of a Rapid Detection Method for Yeast-like Symbionts in Brown Planthopper Based on Droplet Digital PCR Technology

**DOI:** 10.3390/ijms241311071

**Published:** 2023-07-04

**Authors:** Jun Zhang, Chengling Lai, Xuping Shentu, Peiying Hao, Kun Pang, Xiaoping Yu

**Affiliations:** Zhejiang Provincial Key Laboratory of Biometrology and Inspection & Quarantine, College of Modern Science and Technology, China Jiliang University, Hangzhou 310018, China; zhangjun199909@163.com (J.Z.); lchenglai@163.com (C.L.); stxp@cjlu.edu.cn (X.S.); haopeiy@163.com (P.H.)

**Keywords:** droplet digital PCR, YLS, qPCR, insects

## Abstract

The brown planthopper *Nilaparvata lugens* (Stål) (BPH) is a typical monophagous sucking rice pest. Over the course of their evolution, BPH and its symbionts have established an interdependent and mutually beneficial relationship, with the symbionts being important to the growth, development, reproduction, and variation in virulence of BPH. Yeast-like symbionts (YLS), harbored in the abdomen fat body cells of BPH, are vital to the growth and reproduction of the host. In recent research, the symbionts in BPH have mainly been detected using blood cell counting, PCR, real-time quantitative PCR, and other methods. These methods are vulnerable to external interference, cumbersome, time consuming and laborious. Droplet digital PCR (ddPCR) does not need a standard curve, can achieve absolute quantification, does not rely on Cq values, and is more useful for analyzing copy number variation, gene mutations, and relative gene expression. A rapid detection method for the YLS of BPH based on ddPCR was established and optimized in this study. The results showed that the method’s limits of detection for the two species of YLS (*Ascomycetes* symbionts and *Pichia guilliermondii*) were 1.3 copies/μL and 1.2 copies/μL, respectively. The coefficient of variation of the sample repetition was less than 5%; therefore, the ddPCR method established in this study had good sensitivity, specificity, and repeatability. It can be used to detect the YLS of BPH rapidly and accurately.

## 1. Introduction

In recent years, the relationship between insects and their symbiotic microorganisms has become a research hotspot. Symbiotics have been found to contribute to insects’ nutrient metabolism [1,2,3], development [4,5,6,7], reproduction [8,9,10], speciation [11], and improvement of the host’s defenses [12]. The brown planthopper (BPH), *Nilaparvata lugens* (Homoptera: Delphacidae), a distant migratory pest that feeds only on the phloem sap of rice [13], is one of the most seriously damaging pests of rice in China. YLS are dominant endosymbionts in the fatbody of the BPH abdomen [14,15,16]. YLS are closely related to the growth, development, and reproduction of the BPH because YLS provides amino acids, nitrogen storage and recycle, sterol precursors, and vitamin supply for their host [17,18,19,20]. These functions can enable the BPH to exist, feeding solely on rice phloem sap, which is nutritionally imbalanced. In a previous study, the number of YLS in newly emerged female adults of BPH was significantly lower after treatment with methamidophos than in the control group, suggesting that YLS are important in the resistance of BPH [21]. Furthermore, Liu et al. [22] found that Sakuranetin (an antifungal phytoalexin derived from the antibacterial precursor naringenin) can effectively inhibit the growth of YLS. YLS were first isolated by Nasu in 1963, who described their form [23]. Later, Fajun Chen [24] observed four types of symbiotic fungi in BPH using frozen sections and microscopic observation. With the development of molecular biology technology, 18S rDNA, ITS, and 26S rDNA technology are frequently used in microrganism population identification. Symbiotic fungi in BPH were first isolated and identified by Noda et al. [25] using 18S rDNA technology, and they found that YLS were most closely related to *Ascomycotina*. Pang Kun et al. [26] identified *Pichia guilliermondii*, *Cryptococcus peneaus*, and *Candida* using the ITS technique, and verified the presence of these three symbiotic fungi in BPH using nested PCR. Zhang Juefeng et al. [27] cultured two symbionts *Yarrowia lipolytica* and *Sterigmatomyces halophilus* of BPH, obtained using egg block culture with 26S rDNA sequence. According to high-throughput sequencing results [28], *Ascomycetes* symbionts were the most dominant YLS in BPH. In addition, the research group successfully isolated and cultured a strain of *Pichia guilliermondii* in the previous study, so we conducted quantitative detection and analysis for the two YLS.

The most commonly used methods for identifying symbionts are the blood cell counter plate method and qPCR. The former has some limitations, such as the difficulty of distinguishing tiny substances and cells, and some microbial cells being difficult to disperse and easy to miss. The Qpcr relies on the comparison between the amplification curve and the standard curve, which will generate errors in the reaction and conversion process, and low copy number templates are difficult to detect. Unlike Qpcr, droplet digital PCR (ddPCR) analyzes the number of positive droplets rather than the overall fluorescence intensity, so absolute quantification of nucleic acids can also be performed in the absence of known concentrations of standard reference nucleic acids [29,30]. The ddPCR is highly tolerated, significantly reduces inhibitor interference, and can detect templates with low copy numbers. However, because of the inaccuracy of the analysis of nonspecific products, it is necessary to use the probe method with strong specificity. The ddPCR has been widely used in food [31,32], medicine [33], agriculture [34], and other fields because of its high sensitivity, precision, tolerance, and absolute quantification.

The ddPCR technology can be used for absolute quantification of the YLS in BPH without an in vitro culture, which means it can detect the changes in the YLS in BPH quickly and accurately. It is conducive to studying the role of YLS and also provides reference for further exploring new control strategies based on the interaction between symbiotic fungi and host.

## 2. Results

### 2.1. Results of Primer Screening for PCR and RT-qPCR Amplification

**Figure 1:**
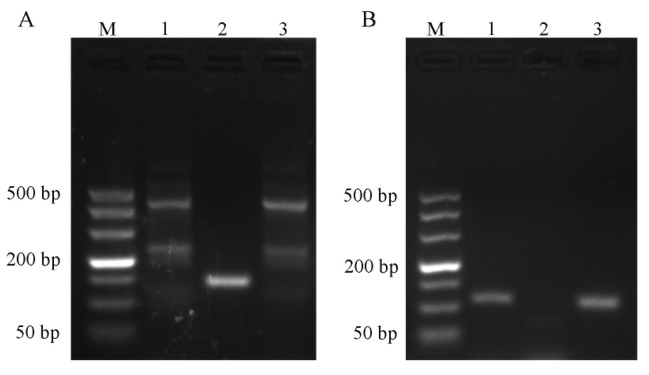
Detection of PCR amplification products. M: DNA molecular weight standard DL 500 Marker; 1, 2, 3 represent three pairs of primers of the same symbionts; (**A**) *Ascomycetes* symbionts; (**B**) *Pichia guilliermondii*.

Electrophoretic results (Figure 1A) showed that only primer No. 2 of *Ascomycetes* symbionts was a single band, which was consistent with the expected length of the amplified product, and so band No. 2 was selected for sequencing. As can be seen in Figure 1B, primers No. 1 and No. 3 of *Pichia guilliermondii* are both single bands, and band No. 3 was selected for sequencing because of its higher brightness.

**Figure 2:**
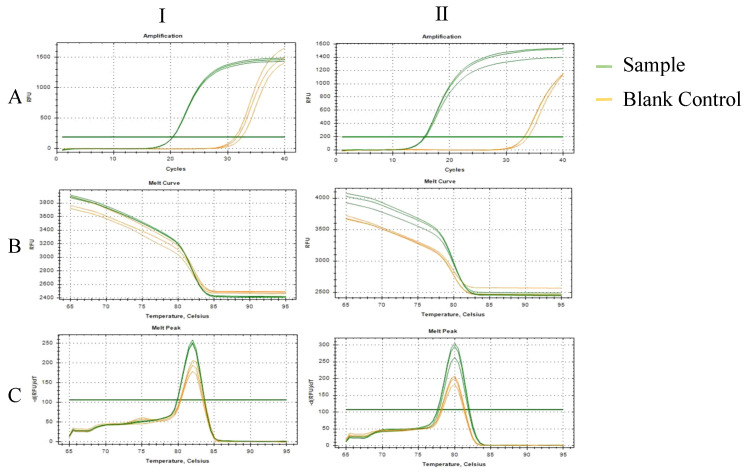
Amplification curve and melting curves of specific primers of two symbiotic fungi of BPH; (**A**): Amplification curve; (**B**): Melt curve; (**C**): Melt curve; Ⅰ: *Ascomycetes* symbionts; Ⅱ: *Pichia guilliermondii*; RFU:Relative Fluorescence Units.

The results of qPCR to verify the specificity of primers (Figure 2A) showed that the amplification curves of the two pairs of primers were all “S”-shaped curves, and the curves did not appear in the blank controls (Figure 2(AⅠ,AⅡ)). The melting curves of the samples were unimodal, which indicates that the two pairs of primers could perform specific amplification (Figure 2B,C).

The specific primers and their corresponding probes of *Ascomycetes* symbionts and *Pichia guilliermondii* were screened based on the experimental results in Section 2.1. The GenBank ID for the primer’s target in *Ascomycetes* symbionts is AF267233.1; the GenBank ID for the primer’s target in *Pichia guilliermondii* is MG601175.1. The specific primers and probe sequences of the two pairs are shown in Table 1. The probe was a FAM-labeled probe.

### 2.2. Optimization of ddPCR System

The optimal annealing temperature was determined by the fluorescence intensity (RFU) amplified by qPCR and the Cq value. The higher the RFU, the lower the Cq value and the optimal annealing temperature were. Therefore, the annealing temperature of the two pairs of primers was selected as 56 °C.

#### 2.2.1. Optimization of Primer Concentration

**Figure 3:**
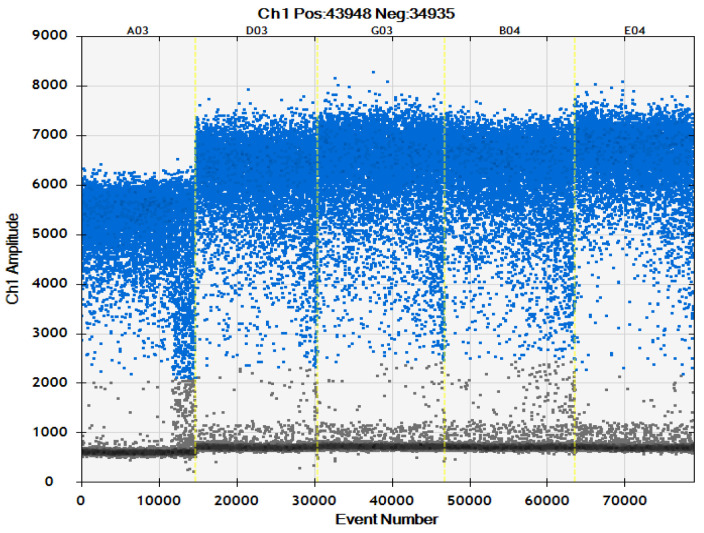
Microdrop scatter map of *Ascomycetes* symbionts ddPCR assay system with different primer concentrations; 600 nM: A03; 700 nM: D03; 800 nM: G035; 900 nM: B04; 1000 nM: E04.

**Figure 4:**
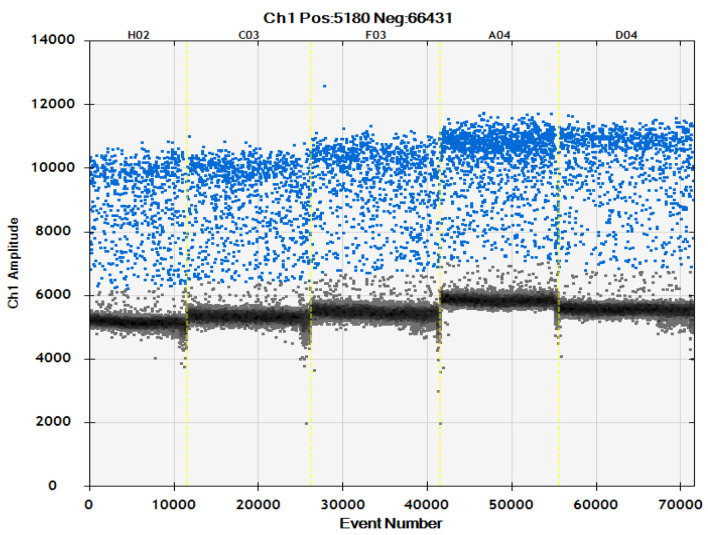
Microdrop scatter map of *Pichia guilliermondii* ddPCR assay system with different primer concentrations; 600 nM: H02; 700 nM: C03; 800 nM: F03; 900 nM: A04; 1000 nM: D04.

This experiment combined optimization of the ddPCR detection system with the results of primer concentration (Table 2, Figure 3 and Figure 4). The primer concentration of *Ascomycetes* symbionts was 800 nM which is recommended concentration of the original system, and that of *Pichia guilliermondii* was 900 nM. There was very little difference between this and other primer concentrations of *Ascomycetes* symbionts, and there was no significant difference in copy number. The copy number in the *Pichia guilliermondii* group with a primer concentration of 900 nM that was the highest, and the 900 nM group was significantly different than the other four groups.

#### 2.2.2. Optimization of Probe Concentration

**Figure 5:**
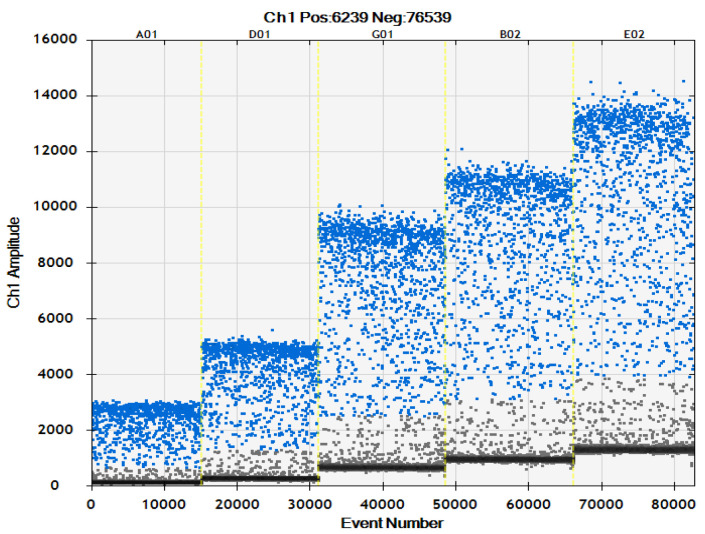
Microdrop scatter map of *Ascomycetes* symbionts ddPCR assay system with different probe concentrations; 62.5 nM: A01; 125 nM: D01; 250 nM: G01; 375 nM: B02; 500 nM: E02.

**Figure 6:**
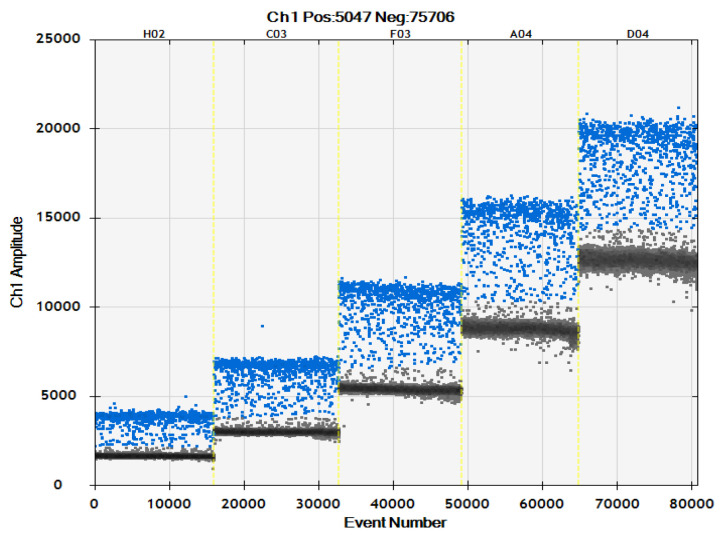
Microdrop scatter map of *Pichia guilliermondii* ddPCR assay system with different probe concentrations; 62.5 nM: H02; 125 nM: C03; 250 nM: F03; 375 nM: A04; 500 nM: D04.

Based on the primer concentration optimization in Section 2.2.1, the probe concentration optimization results of the ddPCR detection system were combined (Table 3, Figure 5 and Figure 6). The probe concentration of *Ascomycetes* symbionts and *Pichia guilliermondii* was 500 nM. When the probe concentration was 500 nM, the positive microdroplet cluster was more obvious, and the positive and negative microdroplets were more obvious.

### 2.3. ddPCR Sensitivity Test

In order to detect the sensitivity of the ddPCR detection system for the two YLS in BPH, the plasmids pMD-As and pMD-Pg were diluted by a 10-fold gradient, and then detected using the established ddPCR detection system. Each concentration of plasmid samples was carried out in triplicate. The results are shown in Table 4 and Table 5. The lowest detection limits of the two detection systems were 1.3 copies/μL and 1.2 copies/μL, respectively, indicating that the sensitivity of the ddPCR detection systems for the two YLS in BPH was high.

### 2.4. ddPCR Specificity Test

The specific results of the ddPCR detection system for *Ascomycetes* symbionts (Table 6) showed that *Ascomycetes* symbionts had specific amplification, while the other four groups of samples were negative. The specificity results of the ddPCR detection system for *Pichia guilliermondii* (Table 7) showed that only *Pichia guilliermondii* showed specific amplification. Therefore, it can be concluded that the rapid detection system of ddPCR established in this study has good specificity.

### 2.5. ddPCR Repeatability Test

The results of the repeatability test (Table 8) show that Related Standard Deviation (RSDS) of the ddPCR assay system for *Ascomycetes* symbionts and *Pichia guilliermondii* was less than 5.00%, and there was no significant difference between the three repeated detection values of the ddPCR detection system for either of the two YLS (*p* > 0.05). This shows that the ddPCR detection system has good repeatability.

## 3. Discussion

The brown planthopper *Nilaparvata lugens* (Stål) is typical monophagous sucking rice pest [13]. It has been found that the YLS in the BPH play a crucial part in its growth [17,35,36,37,38], reproduction [39], and nutrient metabolism [40]. The establishment of a precise and rapid detection method for YLS is conducive to the study of the mutual interaction mechanism between symbiotic fungi and BPH.

In the previous study on the number of symbiotic fungi of BPH, a hemocytometer [41,42] and the qPCR method [43] were used. The hemocytometer is relatively inaccurate due to manual counting, pipetting, and dilution errors. The qPCR also has some limitations [44], such as requiring a standard curve based on known sample concentrations to convert the output data into actual values, and having a low accuracy of quantification that influences the Cq value [45]. Moreover, when Yang et al. [46] compared the results of an experiment enumerating *Cryptosporidium* oocysts in stool samples using ddPCR and qPCR, they found that, within the equal detection range, the results of these two methods were highly linearly and positively correlated with standards (R^2^ ≥ 0.999). However, the precision of ddPCR, as measured by RSD, was better when compared with qPCR [29]. The ddPCR has a higher accuracy than other methods. The rapid detection method based on ddPCR established may be helpful for studying the symbiotic fungi of BPH.

In the area of plant protection, the relationship between herbivorous pests and symbionts has always been the focus of research [47,48]. For example, gut symbionts can influence mating reproduction in the host. In *Bactrocera dorsalis* and *Bactroceracucurbitae* populations, female flies infected with symbiotic microorganisms showed greater attraction to males than those treated with antibiotics to reduce the presence of symbiotic microorganisms [49,50]. Symbionts also can influence the insects’ resistance [51,52]. Additionally, ddPCR technology, as a rapid and efficient method for the detection of insect symbionts, is also widely used for the detection of plant pathogenic bacteria [53,54,55] and insect symbionts [56,57]. When Hickin [58] detected *Wolbachia* in bed bugs using ddPCR, the detection limit was 0.5 copies/μL.

In this study, by screening primers, probes, and optimizing the reaction conditions of the system, we determined that the primers and probe concentrations of the ddPCR rapid detection system for the two YLS, *Ascomycetes* symbionts and *Pichia guilliermondii*, were primers 800 nM, probe 500 nM, and primer 900 nM, probe 500 nM, respectively (Table 2 and Table 3, Figure 3, Figure 4, Figure 5 and Figure 6). Moreover, using the constructed plasmid as a template, the minimum detection limits of ddPCR were 1.3 copies/μL and 1.2 copies/μL, respectively.

Taken together, these data suggest that this ddPCR method is more effective and sensitive for the precise quantification of the YLS. Thus, it can be used as the default method for the subsequent quantitative detection of YLS.

## 4. Materials and Methods

### 4.1. Collection and Culture of Insects

Adult BPH were acquired from rice fields in Hangzhou, China. The BPH populations used in this experiment were established in our laboratory, and these populations were raised on TN1 rice under the following environmental conditions: 25.0 ± 1.0 °C, humidity of 65.0–75.0%, and light: darkness = 16 h:8 h. BPH was fed with rice variety Xiushui 134; the seedbed height was greater than 3 cm, and the seedlings of Xiushui 134 were fed for more than 25 generations.

### 4.2. Specific Experiment

#### 4.2.1. Principal Reagent

TIANNamp Genomic DNA Kit (TIANGEN, Tianjin, China), SanPrep Column DNA Gel Extraction Kit (Sangon Biotech, Shanghai, China), SanPrep Column Plasmid Mini-Preps Kit (Sangon Biotech), Premix TaqTM (TaKaRa TaqTM Version 2.0 plus dye) (TaKaRa, Shiga, Japan), ddPCR related reagents (BIO RAD, Hercules, CA, USA), 10× PBS buffer (Solarbio, Pasig, Philippines).

#### 4.2.2. Main Instruments and Materials

DG8TM Cartridges for QX100TM/QX200TM Droplet Generator (BIO RAD), Droplet Generator DG8TM Gasket (BIO RAD), T100 Thermal Cycler (BIO RAD), Gel DocTM XR^+^ with Image LabTM Software 6.0.1.34 (BIO RAD)

### 4.3. Genome Extraction of Symbiotic Fungi

Thirty newly emerged female BPH adults were prepared, sterilized with 75% alcohol for three times for 3 min, and rinsed with 1 × PBS three times for 3 min. DNA was extracted according to the instructions for the TIANNamp Genomic DNA Kit.

### 4.4. Design and Screening of Primer and Probe

According to the research of high-throughput sequencing results, this study chose to research two YLS of BPH: *Ascomycetes* symbionts and *Pichia guilliermondii*. The sequences were obtained by sequencing, and primers and probes were designed with Beacon Designer. The main principles of primer design were as follows: (a) Primer length: 18–24 bp; (b) Tm: 58–60 °C; (c) Amplified fragment length: 50–150 bp; (d) GC content: 30–80%. The main principles of probe design were as follows: (a) Probe length: 13–30 bp; (b) Tm: 68–70 °C; (c) GC content: 30–80%; (d) The first base at the 5′ end cannot be G; (e) FAM-labeled probe; (f) The second base at the 5′ end cannot be G, etc. The synthesis of primers was by Tsingke Biotechnology Co., Ltd. (Beijing, China). Bands were obtained with PCR and agarose gel electrophoresis, and a single band with higher brightness was selected for sequencing to obtain a single band sequence.

#### 4.4.1. PCR Amplification and Sequencing Alignment

The DNA samples extracted in Section 4.3 were directly used as templates for the amplification reaction. The PCR amplification reaction system were as follows (Total 25 μL): Premix Taq^TM^ for 12.5 μL; dd H_2_O for 9.5 μL; F primer (10 µM) for 1.0 μL; R primer (10 µM) for 1.0 μL; Template DNA for 1.0 μL. The system was prepared in a 200 μL EP tube, and the vortex oscillator was used to shake and mix it. The resulting bubbles were eliminated through brief centrifugation. These tubes were placed into the PCR apparatus for reaction with the following parameters: Initial denaturation: 95 °C, 5 min, cycle reaction; Denaturation: 95 °C, 30 s; Annealing: 56 °C, 30 s; Extension: 72 °C, 10 s for 35 cycles; The last extension: 72 °C, 5 min; Preservation: 12 °C. The heat cap temperature of the PCR apparatus was set at 105 °C and the reaction volume was set at 25 μL. After amplification, 20 μL of PCR products were absorbed and subjected to agarose gel electrophoresis. DNA molecular weight standard DL 500 Marker was selected, and the PCR amplification results were determined on Gel DocTM XR^+^. The sequencing results were compared with the target sequence to determine whether the amplified fragment was the target sequence.

#### 4.4.2. SYBR Green Dye Real-Time Quantitative PCR

The above Section 4.3 extracted DNA samples were directly used as templates for SYBR Green dye real-time quantitative PCR. The reaction systems were as follows (Total 10 μL): 2× SYBR Green Pro Taq HS Premix for 5.0 μL; dd H_2_O for 3.6 μL; F primer (10 µM) for 0.2 μL; R primer (10 µM) for 0.2 μL; Template DNA for 1.0 μL. The reaction system was prepared in the 384-well plate suitable for the CFX384TM real-time quantitative PCR instrument. The membrane was sealed and briefly centrifuged to eliminate bubbles and liquid on the tube wall. The reaction was carried out under the following conditions: Initial denaturation: 95 °C, 30 s, Cycle reaction; Denaturation: 95 °C, 5 s; Annealing: 56 °C, 30 s for 40 cycles. The 384-well plate was then put into a CFX384TM real-time quantitative PCR instrument for the amplification reaction, and the specificity of the primer was determined according to the Melt Curve.

#### 4.4.3. Optimization of Annealing Temperature Using Taq Man Probe Real-Time Quantitative PCR

The DNA samples extracted in Section 4.3 were directly used as the templates for Taq Man probe RT-qPCR. The reaction systems were as follows (Total 10 μL): 2 × AceQ Universal U^+^ Probe Master Mix V2 for 5.0 μL; dd H_2_O for 3.5 μL; F primer (10 µM) for 0.2 μL; R primer (10 µM) for 0.2 μL; probe (10 µM) for 0.1 μL; Template DNA for 1.0 μL. The reaction system was prepared in a 384-well plate, the film was sealed, and it was then briefly centrifuged. The reaction conditions were as follows: Contamination digestion: 37 °C, 2 min; Initial denaturation: 95 °C, 5 min, Cycle reaction; Denaturation: 95 °C, 10 s; Annealing: - °C, 30 s for 40 cycles. The 384-well plate was then placed into a CFX384TM real-time quantitative PCR instrument for amplification reactions, and the annealing temperature was set from 50 to 65 °C according to the Tm of the primer, so as to determine the optimal annealing temperature for the two YLS.

### 4.5. Plasmid Sample Preparation

Using the genome extracted in Section 4.3 as the template, the target fragment was amplified using PCR. The primers used in the PCR reaction are shown in Table 1. The PCR reaction conditions are shown in Section 4.4.1. After amplification, 20 μL of PCR products were extracted and subjected to agarose gel electrophoresis. DNA molecular weight standard DL 500 Marker was selected, and the PCR amplification results were determined on Gel Doc^TM^ XR^+^. If a single band was present, then the target band was recovered using the Test Kit. The recovered product of the target fragment was connected with the pMD™ 19-T Vector Cloning Kit (Sangon Biotech GeneBank ID: MF927778.1), and the ligated product was transformed into DH5α-competent cells. After the positive plasmid in the resistant plate was selected and expanded, the plasmid was sent to Tsingke Biotechnology Co., Ltd. (Hangzhou, China) for sequencing. The obtained positive plasmids were named pMD-As (*Ascomycetes* symbionts) and pMD-Pg (*Pichia guilliermondii*). Plasmids were extracted using the Mini-Preps Kit (Sangon Biotech), and the concentration of the extracted plasmid was determined using NanoDrop 2000. The copy number formula (copies/μL) = 6.02 × 10^23^ copies/mol × concentration (ng/μL)/(number of bases × 660 g/mol) × 10^−9^. Plasmids were diluted to specific concentrations using a DNase/RNase-Free Water (Solarbio) gradient.

### 4.6. ddPCR System Optimization

Using the gradient dilution plasmid samples in Section 4.5 as templates, ddPCR reaction was carried out with the primers and probes obtained from screening, and the experimental procedures were as follows: the ddPCR reaction system was prepared according to the standard system, and the reaction systems were as follows (Total 20 μL): ddPCR^TM^ Supermix for Probe (No dUTP) for 10 μL; dd H_2_O for 4.9 μL; F primer (10 µM) for 1.8 μL; R primer (10 µM) for 1.8 μL; probe (10 µM) for 0.5 μL; Template DNA for 1.0 μL. The samples were mixed by shaking and centrifugation briefly. After all samples were added into the 96-well plate, the sealing film was placed on the 96-well plate and fixed (red line marking face up). The pre-heated PX1 sealing device was used to seal the 96-well plate. The operation was performed at 180 °C, for 5 s; then, the PCR amplification reaction was performed in a 96-well PCR apparatus. The reaction conditions were as follows: Initial denaturation: 95 °C, 10 min; cycle reaction, Denaturation: 94 °C, 30 s; Annealing: 56 °C, 1 min for 40 cycles; The last extension: 98 °C, 10 min; Preservation: 4 °C. The heat cap temperature of the PCR apparatus was set at 105 °C, and the reaction volume was set at 40 μL.

### 4.7. Sensitivity Test

To assess the sensitivity of the rapid detection method based on ddPCR for the symbiotic fungi *Ascomycetes* symbionts and *Pichia guilliermondii* of BPH, the pMD-As and pMD-Pg plasmids constructed in Section 4.5 were used for gradient dilution, and then qPCR and ddPCR were used to detect the gradient dilution plasmid samples. The minimum detection limit of the ddPCR method was determined, and the sensitivity of the two methods for the detection of plasmid samples was compared.

### 4.8. Specific Test

Based on the previous high-throughput sequencing results, we selected two symbiotic bacteria in the BPH, *Arsenophonus* symbionts and *Acinetobacter soli* (*A. soli*). The sequence was obtained by sequencing, the GeneBank IDs are KM593930.1 (*Arsenophonus* symbionts) and MT394056.1 (*A. soli*), and the primers and probes were designed with Beacon Designer. The main principles of primer design are shown in Section 4.4. The specific primers and probe sequences of the two pairs are shown in Table 9. Bands were obtained via PCR and agarose gel electrophoresis, and a single band with high brightness was selected for sequencing to obtain a single-band sequence. DNAMAN software was used to compare whether the sequence obtained by sequencing matched the target sequence, and the amplified fragment of primer was determined as the target sequence. Standard plasmids of *Arsenophonus* symbionts and *Acinetobacter soli* (*A. soli*) were prepared according to the steps in Section 4.5. They were named pMD-Ar (*Arsenophonus* symbionts) and pMD-Acs (*Acinetobacter soli* (*A. soli*). The optimized ddPCR system was used to detect the above two plasmids, and the specificity of the ddPCR method in this experiment was evaluated.

### 4.9. Data Statistics and Analysis

Statistical data analysis and plotting were performed using SPSS Statistics and GraphPad Prism 6.01. Data are expressed as mean ± standard deviation. The difference in mean value was analyzed using one-way analysis of variance (ANOVA) and Tukey’s multiple comparisons, with the statistical significance of the difference set at *p* < 0.05.

## 5. Conclusions

The ddPCR method established in this study has high sensitivity, specificity, and repeatability. It can be used to detect the YLS of BPH rapidly and accurately.

## Figures and Tables

**Table 1:** Specific primers and probe sequences of two YLS.

Symbionts	Primers	Sequences (5′ → 3′)	Amplification Product Size (bp)
*Ascomycetes* symbionts	AsF	GTCGTAGTCTTAACCATAA	145
	AsR	CTTCCGTCAATTTCTTTAAG	
	AsP	TCAGCCTTGCGACCATA	
*Pichia guilliermondii*	PgF	CCTCTCAATGTATTAGGTTTA	101
	PgR	TGAGGTCAAACTTGTTTG	
	PgP	CCAACAATACCAGAAATATCCCGCC	

Note: Both YLS probes were modified with 6-FAM at the 5′ end and BHQ-1 at the 3′ end.

**Table 2:** The copy number of the ddPCR detection system for YLS (primer concentration).

Symbionts		Primer Concentration (nM)				
		600	700	800	900	1000
*Ascomycetes* symbionts	Copy number copies/μL	903.00	962.00	965.00	963.00	965.00
*Pichia guilliermondii*	Copy number copies/μL	73.00	80.00	79.60	134.00	76.00

**Table 3:** Copy number of the ddPCR detection system for YLS (probe concentration).

Symbionts		Probe Concentration (nM)				
		62.5	125	250	375	500
*Ascomycetes* symbionts	Copy number copies/μL	81.10	89.00	100.00	92.20	97.00
*Pichia guilliermondii*	Copy number copies/μL	79.20	78.90	74.90	68.80	77.50

**Table 4:** Copy number of the ddPCR detection system for *Ascomycetes* symbionts (sensitivity).

Dilution Ratio	ddPCR/(copies·μL^−1^)		
	Result 1	Result 2	Result 3
10^−5^	401.00	468.00	630.00
10^−6^	42.90	46.40	54.00
10^−7^	9.30	10.30	13.60
10^−8^	1.80	1.70	1.50
10^−9^	1.30	1.40	No call

**Table 5:** Copy number of the ddPCR detection system for *Pichia guilliermondii* (sensitivity).

Dilution Ratio	ddPCR/(copies·μL^−1^)		
	Result 1	Result 2	Result 3
10^−5^	475.00	464.00	520.00
10^−6^	40.90	84.00	85.00
10^−7^	7.70	9.10	9.70
10^−8^	4.60	5.90	6.90
10^−9^	1.70	No call	1.20

**Table 6:** Copy number of the ddPCR detection system for *Ascomycetes* symbionts (specificity).

Sample	*Ascomycetes* Symbionts	*Arsenophonus* Symbionts	*Acinetobacter soli*	*Pichia* *guilliermondii*	ddH_2_O
Copy number copies/μL	20.50	2.60	1.70	3.60	0.80
	22.10	1.40	1.40	5.00	0.65
	23.10	1.80	1.80	5.30	No call

**Table 7:** Copy number of the ddPCR detection system for *Pichia guilliermondii* (specificity).

Sample	*Ascomycetes* Symbionts	*Arsenophonus* Symbionts	*Acinetobacter soli*	*Pichia* *guilliermondii*	ddH_2_O
Copy number copies/μL	2.20	2.40	0.66	64.20	0.56
	1.50	No call	0.90	63.00	0.80
	2.00	1.70	1.30	71.00	0.90

**Table 8:** Repeatability of the ddPCR detection system for two YLS.

Symbionts	N	Average Value (copies/μL)	Standard Deviation (copies/μL)	Relative Standard Deviation (%)	Results of One-Way ANOVA
*Ascomycetes* symbionts	1	84.00	2.65	3.15	F = 1.915 *p* = 0.227
	2	80.00	2.00	2.50	
	3	76.33	2.52	3.30	
*Pichia guilliermondii*	1	35.67	1.53	4.28	F = 1.126 *p* = 0.385
	2	35.50	0.53	1.49	
	3	36.62	0.54	1.47	

**Table 9:** Specific primers and probe sequences of two symbiotic bacteria.

Symbiotic Bacteria	Primer	Sequence (5′-3′)	Length of Amplified Product (bp)
*Arsenophonus* symbionts	ArF	GGGAATATTGCACAATGG	125
	ArR	CGTCAATTGCTAAGGTTA	
	ArP	AACCTTAACACCTTCCTCACGACT	
*Acinetobacter soli*	AcF	GCCAATTAAGTCAAATGTG	102
	AcR	GCTACACCTGGAATTCTA	
	AcP	CCACACTCTAGCCAACCAGTATCG	

Note: Both bacterial probes were modified with 6-FAM at the 5′ end and BHQ-1 at the 3′ end.

## Data Availability

All data are included in figures, or can be obtained by contacting the corresponding author.
